# Sensing the restoring force characteristics for detached house structural health monitoring using a camera and accelerometer fusion

**DOI:** 10.1038/s41598-025-88949-7

**Published:** 2025-02-11

**Authors:** Shun Muramatsu, Keito Tamura, Michitaka Yamamoto, Seiichi Takamatsu, Toshihiro Itoh

**Affiliations:** 1 Department of Precision Engineering, Graduate School of Engineering, The University of Tokyo, Tokyo, Japan; 2 Department of Electrical Engineering, Faculty of Engineering, Tokyo University of Science, Tokyo, Japan; 3 School of Systems Science and Industrial Engineering, Thomas J. Watson College of Engineering and Applied Science, State University of New York at Binghamton, Binghamton, NY USA

**Keywords:** Structural health monitoring, Restoring force characteristics, Camera, Accelerometer, Civil engineering, Electrical and electronic engineering

## Abstract

A new method to sense the restoring force characteristics using camera and accelerometer fusion was developed. Simple methods for monitoring detached wooden houses are required to minimize earthquake damage. The restoring force characteristics, which are calculated using the interstory drift and response acceleration, must be measured. However, the conventional method requires a complex system to synchronize multiple accelerometers and suffers from acceleration integration errors. In this study, a method that uses a camera in addition to an accelerometer was developed. The interstory drift was measured by a camera, and the response acceleration was calculated by adding the ground acceleration measured by the accelerometer and the interstory acceleration. These can be used to obtain the restoring-force characteristics of a house when assuming a single-mass system. In addition, this method has no integration errors as those in previous studies. The scale model experiments demonstrated that the restoring force characteristics could be measured with a measurement error of 1% using a common camera. A shake table experiment demonstrated that the prototype fabricated utilizing the camera and accelerometer successfully measured the restoring force characteristics of a full-scale two-by-four timber-framed building.

## Introduction

Since ancient times, earthquakes have significantly impacted people’s livelihoods. For example, the 2010 Haiti earthquake^1^, 2011 Tohoku earthquake and tsunami^2^, and 2016 Kumamoto earthquake^3^. As a result, preparation for earthquakes is becoming increasingly important. A key issue is the diagnosis of damage caused by earthquakes^4^. This is particularly important for detached wooden houses in Japan^5^. Therefore, research on structural health monitoring of detached wooden houses is gaining momentum^6^.

Structural health monitoring can be broadly divided into two types: characteristics and responses. Characteristic monitoring is used to evaluate the characteristics of a building, including its vibrational properties, stiffness, and damping. It can assess the structural integrity of a building based on changes before, during, and after an earthquake^7–9^. For example, it has been reported that when an earthquake damages structural parts, the stiffness and natural frequency of the building decrease^7,8^. However, natural frequencies are affected by other factors such as aging, temperature, and humidity^10^. Therefore, using natural frequencies to assess building damage is difficult. In contrast, response monitoring evaluates a building’s response to external forces such as earthquakes and wind. It is based on acceleration, interstory drift, and other building responses and can directly evaluate the damage caused by earthquakes.

As a response monitoring method for high-rise buildings, the interstory drift angles of each layer are measured by attaching an accelerometer to each floor^11^. The interstory drift angle is obtained by dividing the interstory drift between building by the height between the layers. It can assess the damage to a building by comparing it with a threshold value established by preliminary structural analysis. However, the requirement for a preliminary structural analysis is impractical for adapting to individual houses already built. In addition, it has been reported that large displacements of buildings cause large errors when this method is used^12^. To overcome these problems, methods that use restoring force characteristics for structural health monitoring have attracted attention in recent years^13,14^. The restoring force refers to the force that causes a building to return to its original shape when it is deformed, for example, by shaking, as in an earthquake. When the deformation of the building is relatively small, that is, when the deformation is within the elastic range, the building recovers completely. In contrast, when large displacements cause damage to the building, that is, the deformation is within the inelastic range, permanent deformation remains and the restoring force becomes smaller. Therefore, the extent of damage can be determined by measuring the characteristics of the restoring force against an earthquake and checking whether the displacements are within the elastic or inelastic range.

The restoring force characteristics can be obtained from the response acceleration and interstory drift. To achieve this, a method using multiple accelerometers has been proposed^13,14^. In this method, accelerometers are attached to the first and second floors. The response acceleration corresponded to the accelerations of the second floor, and the interstory drift was calculated by integrating the difference between the two accelerations. However, this conventional technology requires a highly accurate time synchronization of the accelerometers attached to each layer. This requires a complex and expensive system that is difficult to adapt to individual houses. Furthermore, the interstory drift is calculated by integrating the acceleration twice, which results in integration errors, and there are still measurement restrictions^14^; therefore, additional methods are required to measure the interstory drift of individual houses accurately.

To measure interstory drift, displacement memory sensors^15^, laser displacement transducers^16^, GNSS (Global Navigation Satellite System) displacement transducers^17^, optical position sensors^18^, and camera-based methods^19–23^ have been proposed. However, displacement memory sensors, laser displacement transducers, and GNSS displacement transducers must be installed and synchronized with multiple sensors, which is still problematic for individual houses. Optical position sensors require installation of an LED light source on the ceiling. For camera-based methods, displacement measurement methods have been researched for structural monitoring, such as railway bridge^24–26^, building^19–23^, and various other structures^27^, have been researched for structural monitoring. The studies on buildings, in particular, have applied methods to measure interstory drift and successfully demonstrated them. However, these camera-based methods cannot obtain the restoring force characteristics because the response acceleration cannot be measured using only a camera.

In this study, we propose a new and simple measurement method to obtain restoring force characteristics using a camera and an accelerometer. The interstory drift is measured using a camera. The response acceleration, which could not be measured by the camera alone, was calculated by adding the ground acceleration measured by the accelerometer to the interstory acceleration measured by the camera. Furthermore, these instruments can be used to obtain the restoring-force characteristics of a house when a single-mass system is assumed. The proposed method can directly measure displacement using a camera, eliminating the need to integrate acceleration and avoiding integration errors. It can also eliminate synchronization difficulties because the camera and accelerometer are wired, connected to each other, and run on the same clock. Furthermore, the proposed device can calculate the response acceleration, which is difficult to calculate in the conventional form. In this study, the concept of the proposed method is verified using a small-scale model, and the effect of camera sampling rate on the proposed method is evaluated. In addition, a compact prototype device consisting of a camera and accelerometer is fabricated, and the restoring force characteristics of a full-scale two-by-four wood-framed building is demonstrated using the fabricated device.

## Proposed method

**Fig. 1:**
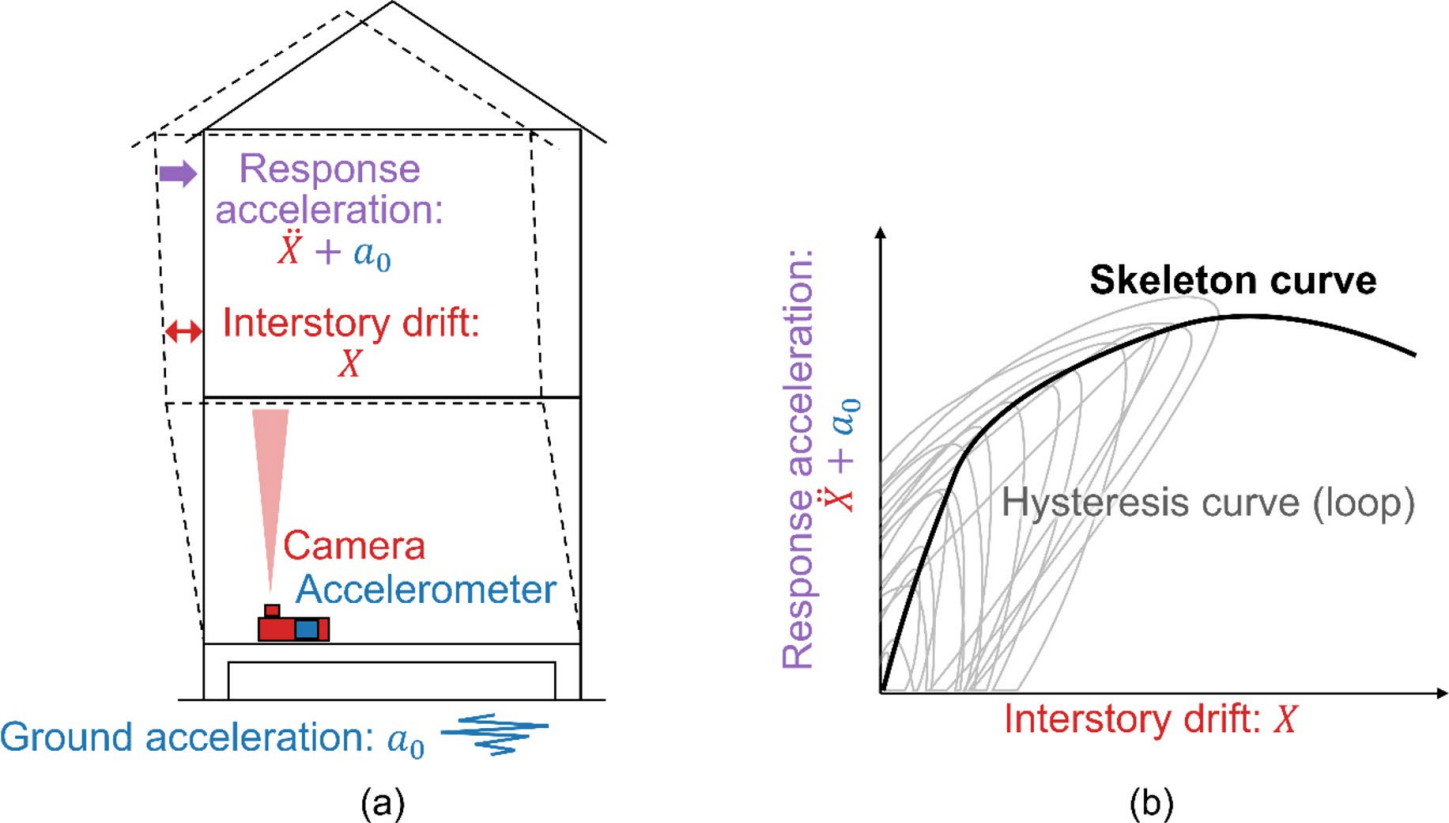
Proposed method capable of measuring the restoring force characteristics using a camera and an accelerometer. (**a**) Schematic image of measuring the interstory drift and response acceleration with the camera and accelerometer. (**b**) Restoring force characteristics calculated with the measured interstory drift and response acceleration.

The proposed method is illustrated in Fig. 1. This method aims to obtain a skeleton curve, which is a restoring force characteristic, using a camera and an accelerometer. Figure 1 (a) shows an overview of the measurement setup. A camera and accelerometer are installed on the first floor. The accelerometer measures the ground acceleration and is used to calculate the response acceleration. The camera measures the interstory drift directly and is used to calculate the response acceleration. The proposed method considers a detached house as a single-degree-of-freedom (SDOF) model according to previous studies^28–30^. The section comprising the second floor and above is considered the mass, and the first floor is considered the ground. Therefore, the displacement of the mass in the SDOF model corresponds to the interstory drift between the first and second floors. In general, the equation of motion for a single-mass system is expressed as follows:

1 $$m\ddot{X}+Q=-m{a}_{0}.$$

where $\:Q$ denotes the restoring force of the building, $\:X$ denotes the interstory drift between the first and second floors, $\:\ddot{X}$ denotes twice the derivative of $\:X$, $\:{a}_{0}$ denotes the ground acceleration, and $\:m$ denotes the building mass. Therefore, the response acceleration corresponding to the restoring force can be expressed as

2 $$\frac{Q}{m}=-\left({a}_{0}+\ddot{X}\right).$$

This implies that the restoring force characteristics can be expressed as the relationship between the interstory drift $\:X$ and response acceleration $\:\ddot{X}+{a}_{0}$.

An image of the restoring force characteristics is shown on Fig. 1 (b). The relationship between the interstory drift and the response acceleration during the vibration, for example, the earthquake, draws the hysteresis curve (hysteresis loop). The skeleton curve can be calculated using the hysteresis curve and indicates the degree of building damage^14,31^. For example, the linear section of the skeleton curve indicates no damage, whereas the nonlinear section indicates minor or moderate damage, and the section with negative stiffness indicates significant damage.

## Experimental methods

### Principle verification using a scale model

**Fig. 2:**
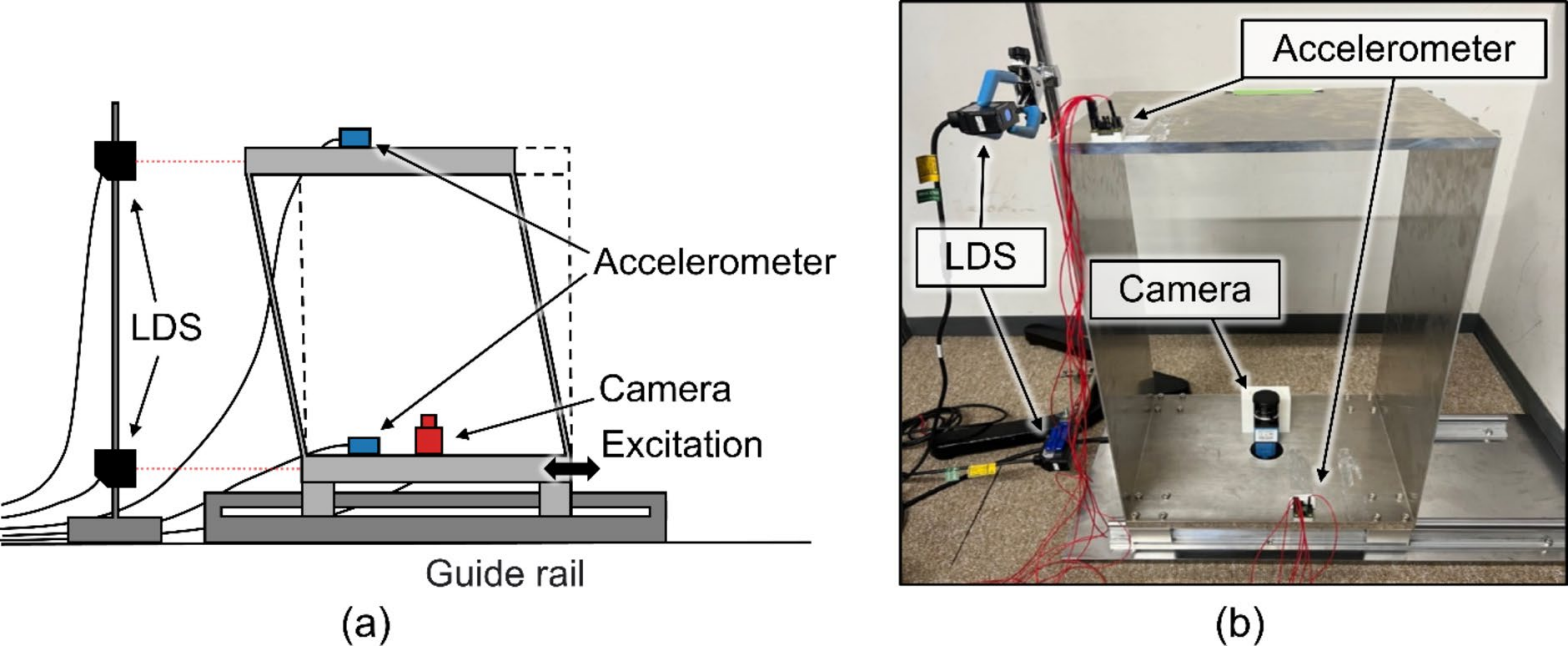
Experimental setup with the scale model. (**a**) Schematic image. (**b**) Actual setup.

**Fig. 3:**
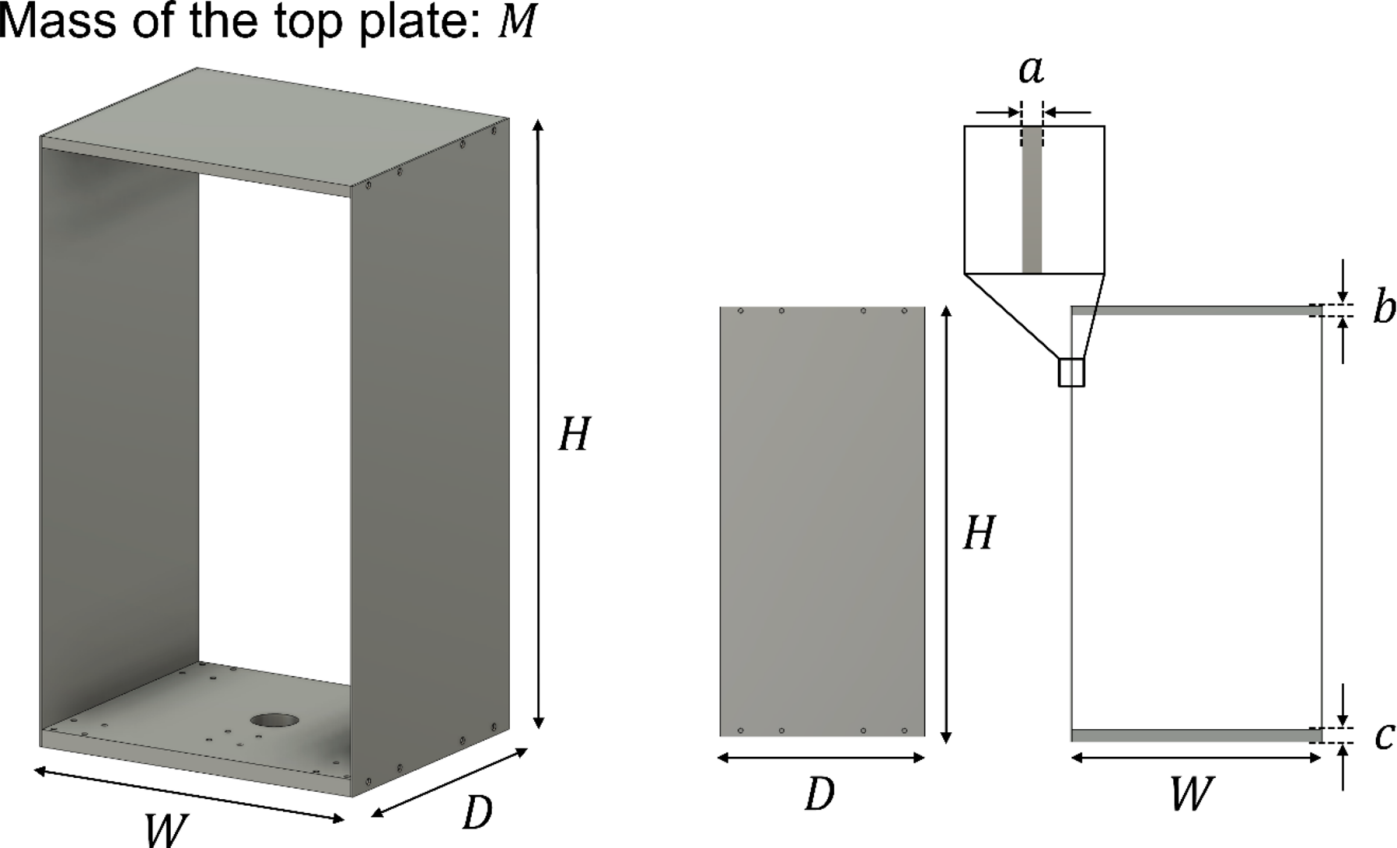
Geometric parameters of the scale model.

A scale model was prepared to test the proposed method for measuring the restoring force characteristics using a camera and an accelerometer. The experimental setup for the prepared scale model is shown in Fig. 2. The model could be deformed along only one axis because it was installed on linear guide rails. The details of each model parameter are presented in Fig. 3; Table 1. The designed natural frequency of the model was 5.33 Hz to conform to that of a typical wooden house (5–7 Hz). This value was verified using finite element analysis (COMSOL Multiphysics, COMSOL Inc., Stockholm, Sweden). The resulting value was 5.2864 Hz, which was reasonable because the order matched the designed value. To evaluate the proposed method, a camera (DMK33UX273, Imaging Source Europe GmbH, Bremen, Germany) with a camera lens (VS-2518VM, VS Technology Corporation, Tokyo, Japan) and an accelerometer (EVAL-ADXL354CZ, Analog Devices, Wilmington, MA, USA) were attached to the underside of the model. A marker was attached to the ceiling to calculate the displacement measured using the camera. As a reference, an accelerometer was mounted on the ceiling side of the model, and laser displacement sensors (LDS) (IL-S100, KEYENCE CORPORATION, Osaka, Japan) were mounted on the upper and lower sides of the model. In the response acceleration was calculated from the accelerometer on the ceiling side, and the interstory drift was calculated from the difference in the LDS. The frame rate of the camera was 100 fps and the values were acquired using a data logger (NI-9206, National Instruments Corporation, Austin, TX, USA). The sampling rates of the accelerometer and LDS were set to approximately 600 Hz.

**Table 1:** Parameters of the scale model.

*M* (kg)	*W* (mm)	*D* (mm)	*H* (mm)	Young’s modulus: *E* (GPa)	Moment of inertia of area: *I* (m^4^)
2	300	250	500	70	1.66667 × 10^− 10^

*a* (mm)	*b* (mm)	*c* (mm)	Rigidity of the plate: *k* (N/m)	Natural frequency: *f* (Hz)	Amount of deformation: *Δ* (mm)
2	10	15	2240	5.33	8.75

**Fig. 4:**
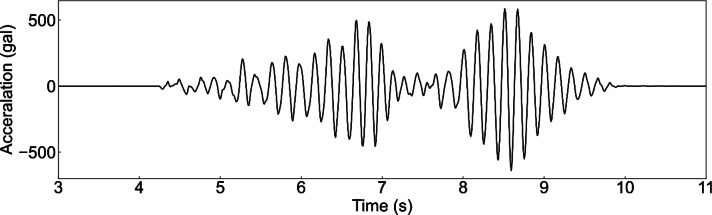
Input vibration signal applied to the scale model.

The model was vibrated to simulate earthquakes. Figure 4 shows the input vibration signal as acceleration. The response acceleration and interstory drift were determined using the proposed method. The displacement measurement method from the camera images was based on a modified kernelized correlation filter algorithm, which is a region-tracking method^32^. Moreover, the restoring force characteristics were acquired using interstory drift and response acceleration. The response displacement, response acceleration, and restoring force characteristics obtained using the proposed method were compared with the reference results.

Because the frame rate of 100 fps is faster than that of a normal camera, the effect of varying the frame rate of the camera to 40, 60, 80, and 100 fps was evaluated to assess whether measurements could be performed using cameras with more common and slower frame rates. Furthermore, correction by upsampling was verified to ensure accuracy, including when using cameras with slower frame rates. Upsampling was performed using cubic spline interpolation.

### Shake table experiment for a wooden house with a fabricated device

**Fig. 5:**
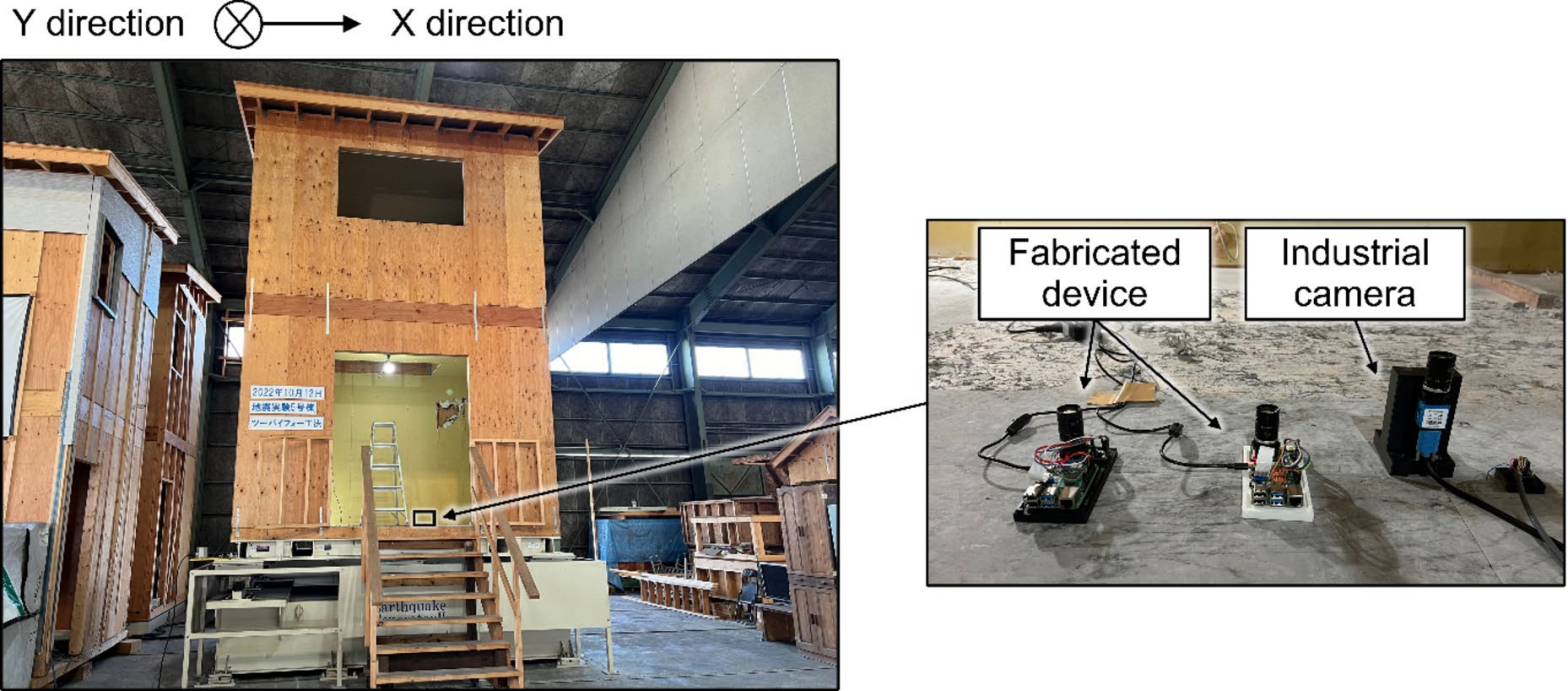
Experimental setup of the fabricated compact devices on the shake table.

Based on the results of experiments using the scaled model, a compact device was fabricated to apply the proposed method to actual houses. The device included an accelerometer (EVAL-ADXL355Z, Analog Devices, Wilmington, MA, USA), a camera module (Raspberry Pi Global Shutter Camera, Raspberry Pi Ltd., Cambridge, England) with a camera lens (VS-5026VM, VS Technology Corporation, Tokyo, Japan), and a single-board computer (Raspberry Pi 4, Raspberry Pi Ltd., Cambridge, England). The device is fabricated from PLA (polylactic acid) using a 3D printer. The fabricated device was evaluated in a shake table experiment for a wooden house. The experimental setup is illustrated in Fig. 5. The wooden house used in the experiment is a full-scale two-by-four timber-framed building with a floor-to-ceiling height of 2450 mm. The fabricated device was attached to the floor and markers were placed on the ceiling to facilitate the measurement of displacement using a camera. A high-performance industrial camera used in the scale model experiment was attached to the floor as a reference, and accelerometers were attached to the floor and ceiling. The house vibrated in a circular pattern with gradual acceleration on the shaking table. The input acceleration was increased to the upper 6 of the Japanese Meteorological Agency’s seismic intensity scale, and the total vibration time was 40–50 s.

The restoring force characteristics calculated from the interstory drift and the response acceleration measured from the fabricated device were evaluated. The interstory drift was compared with that of an industrial camera. To evaluate the restoring force characteristics, the natural frequencies of the entire house were obtained from accelerometers and compared with reference values. This is because the house was significantly large to install an LDS, unlike the experiments with the scaled model, and the low-frequency region of the accelerometer outputs had integral errors, making it difficult to discuss the exact numerical values.

## Results and discussion

### Principle verification using the scale model

**Fig. 6:**
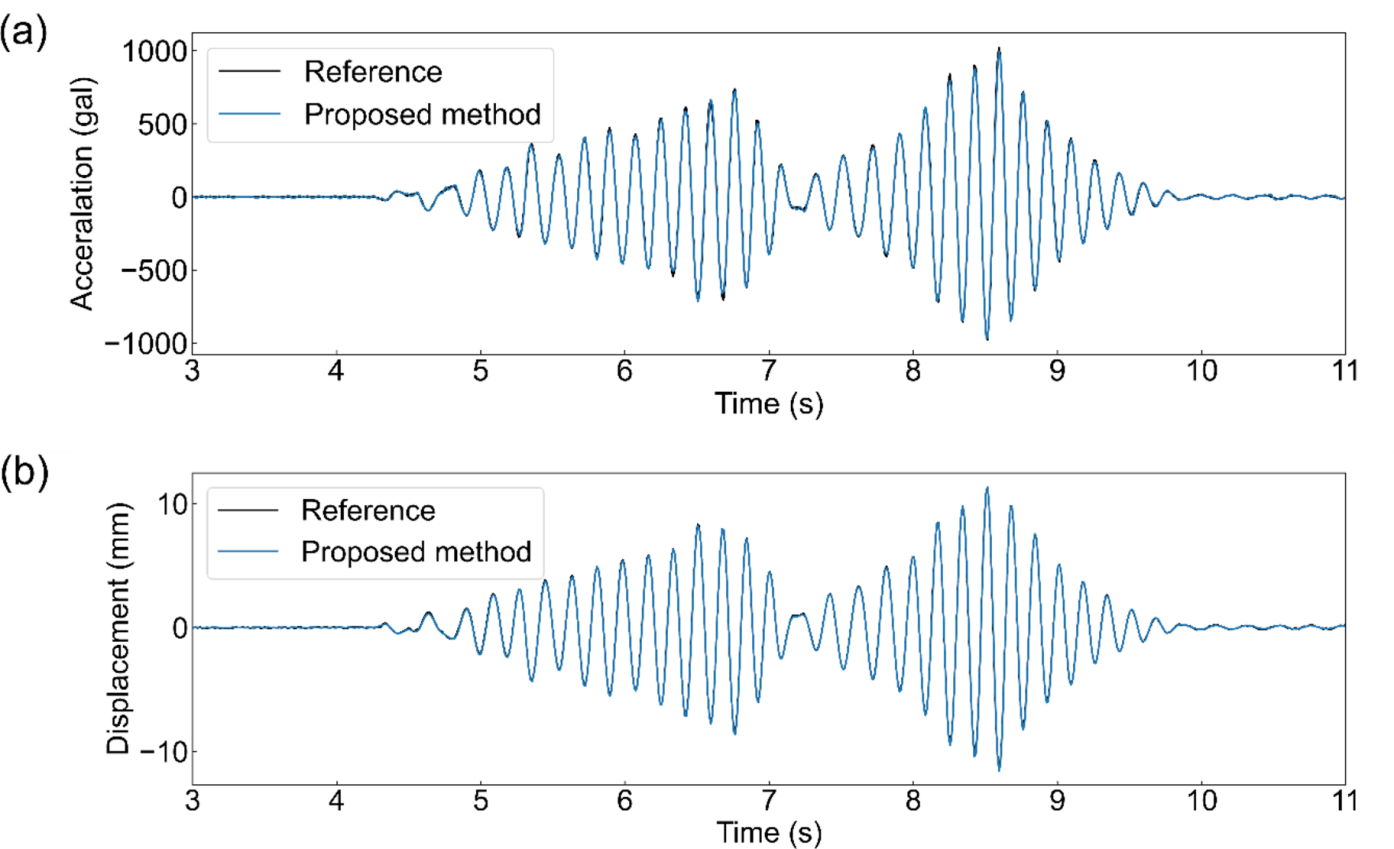
Example of the measured signal. (**a**) Response acceleration measured by the proposed method and the reference. (**b**) Interstory drift measured by the proposed method and the reference.

**Fig. 7:**
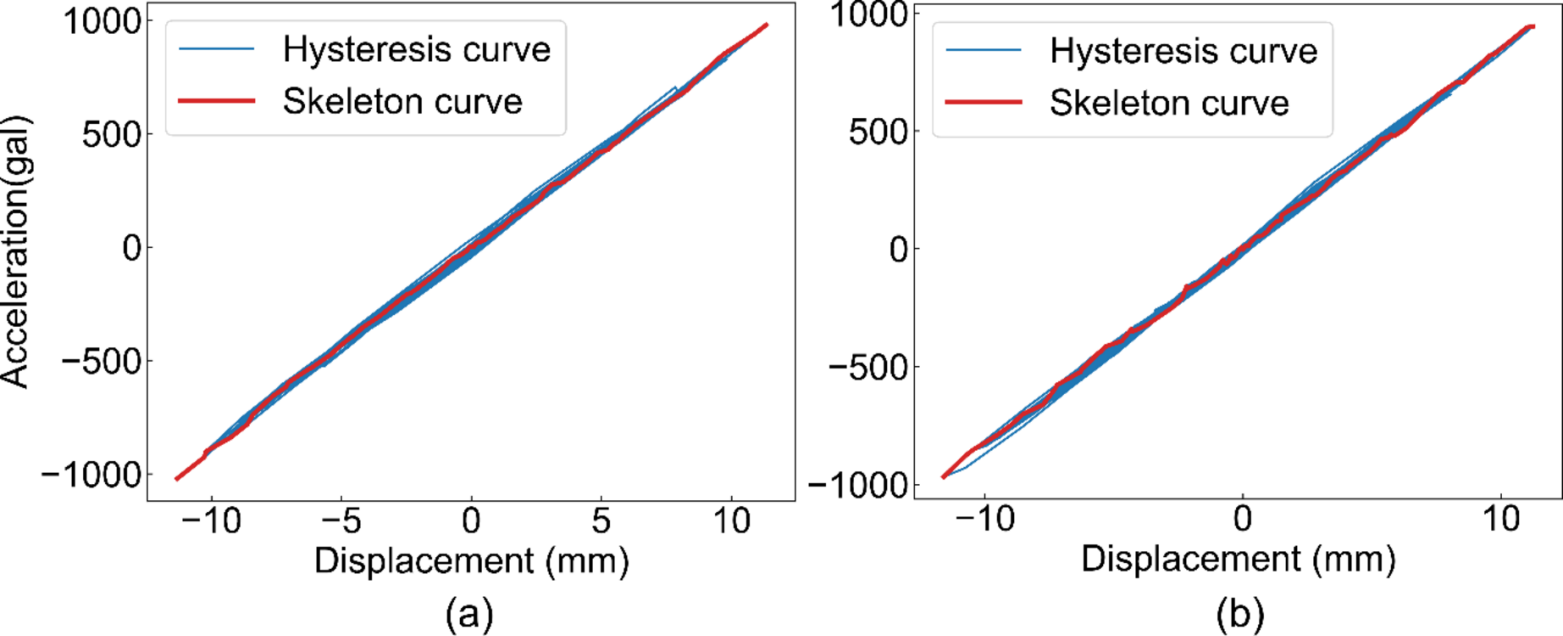
Measured restoring force characteristics. (**a**) Reference method. (a) Proposed method.

Figure 6 (a) shows the measurement results of the response acceleration for each of the proposed method and references when the vibrations shown in Fig. 4 were applied to the scale model. In contrast, Fig. 6 (b) shows the measurement results of the interstory drift. These figures show that signals comparable to the reference were obtained for both the response acceleration and interstory drift. Figure 7 shows the restoring force characteristics plotted from the relationship between the response acceleration and interstory drift using the proposed method and reference. The slope of the skeleton curve was 865 rad^2^/s^2^ for the reference method and 838 rad^2^/s^2^ for the proposed method. This indicates that measurement is possible with an error of approximately 3%. However, the proposed method yielded slightly smaller values; that is the initial stiffness was underestimated.

**Fig. 8:**
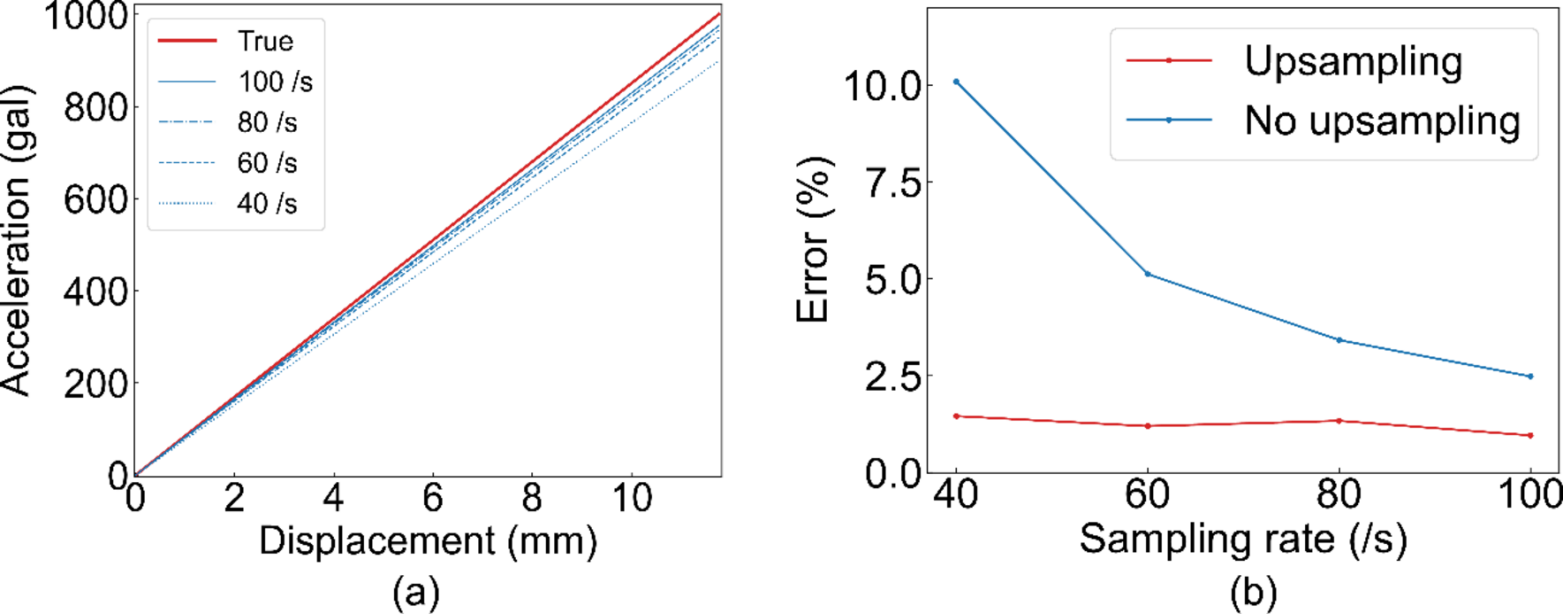
Effect of frame rate of the camera. (**a**) The restoring force characteristic measurement result of varying camera frame rate. (**b**) Error rate against sampling rate (camera flame rate) with and without upsampling.

The underestimation was caused by an error resulting from numerical differentiation. To clarify the influence of errors during the numerical differentiation, the same evaluations were conducted by varying the sampling rate of the camera. Figure 8 (a) shows the change in the regression line of the skeleton curve when the sampling rate changes. This indicated that the slope of the restoring force characteristic was underestimated as the sampling rate decreased. This indicates that the error in differentiation is one of the reasons for underestimation.

Thus, we attempted to upsample each interstory drift value calculated by the camera up to 300 /s. The results of the upsampling are shown in Fig. 8 (b). It shows that upsampling reduced the error by approximately 1%. Therefore, the upsampling process enabled highly accurate interstory drift measurement with an error of 1% including a normal camera at 40 fps.

### Shake table experiment for the wooden house with the fabricated device

**Fig. 9:**
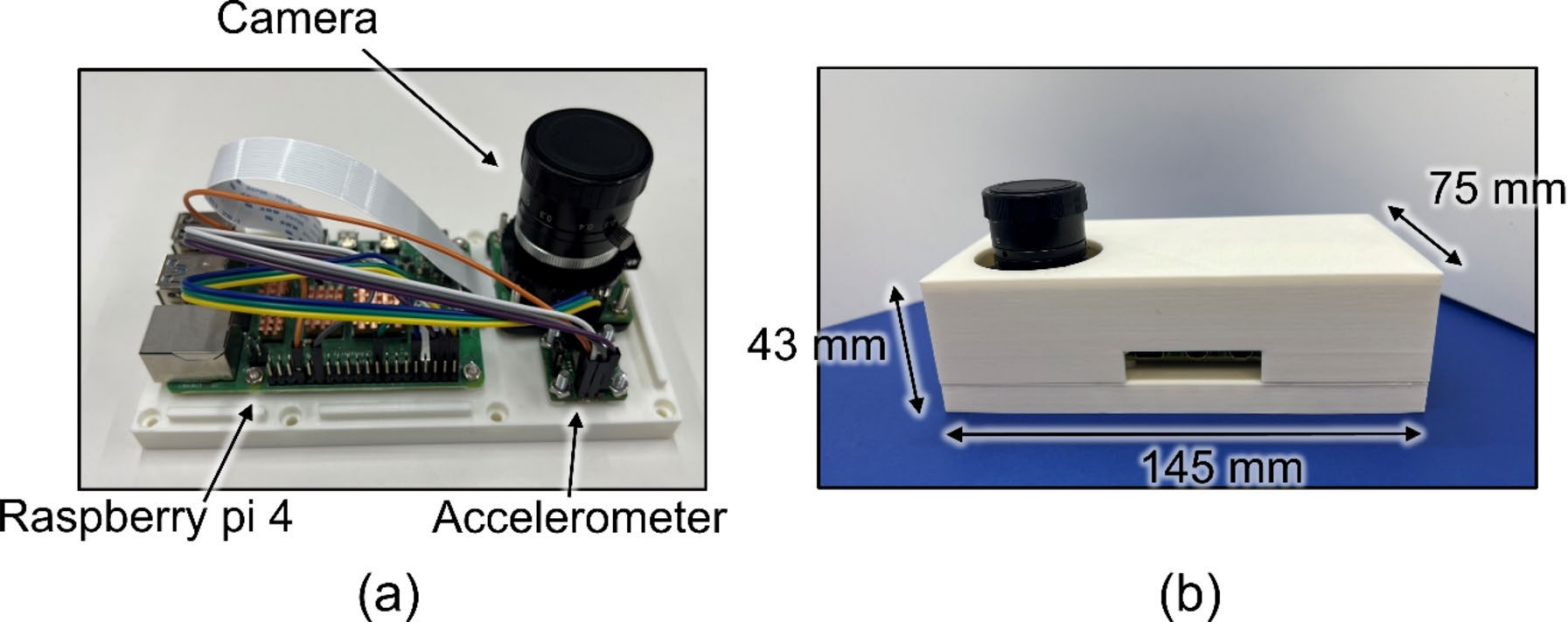
Fabricated compact device for the proposed method. (**a**) Inside of the case. (**b**) Outside of the case.

Figure 9 shows the fabricated compact device. It consists of a single-board computer for data processing, a camera, and an accelerometer for measurement and is packaged within 75 mm × 145 mm × 43 mm. Owing to its compact size, it is expected to be easily installed in private houses.

**Fig. 10:**
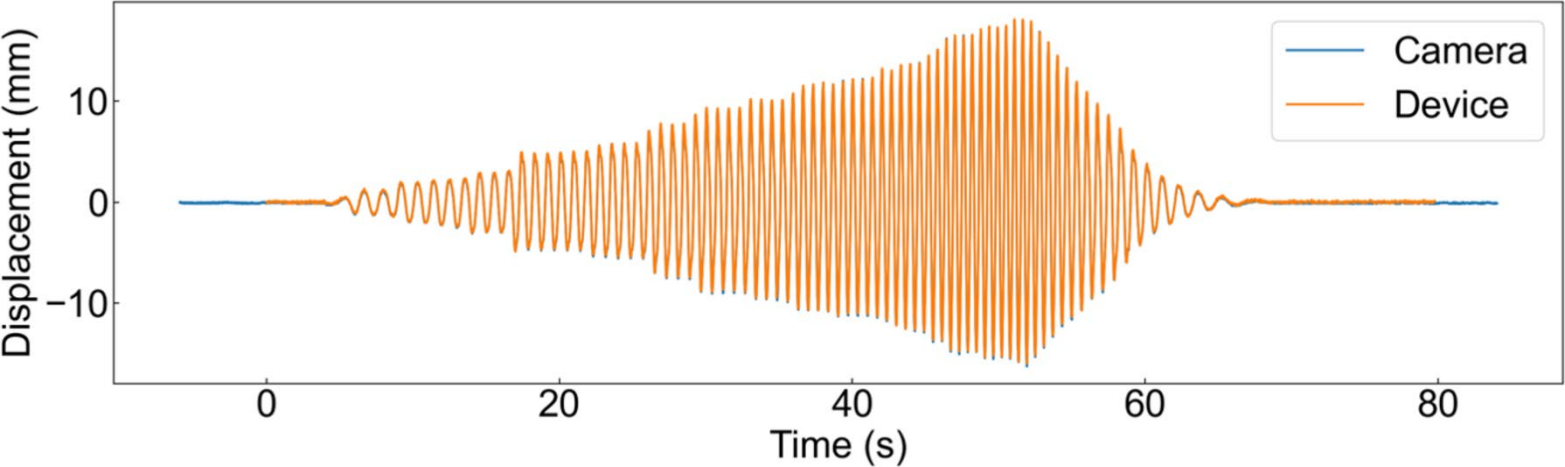
Measurement results of the interstory drift of X direction using the camera of the fabricated module and the higher-performance industrial camera during vibration table experiment.

**Fig. 11:**
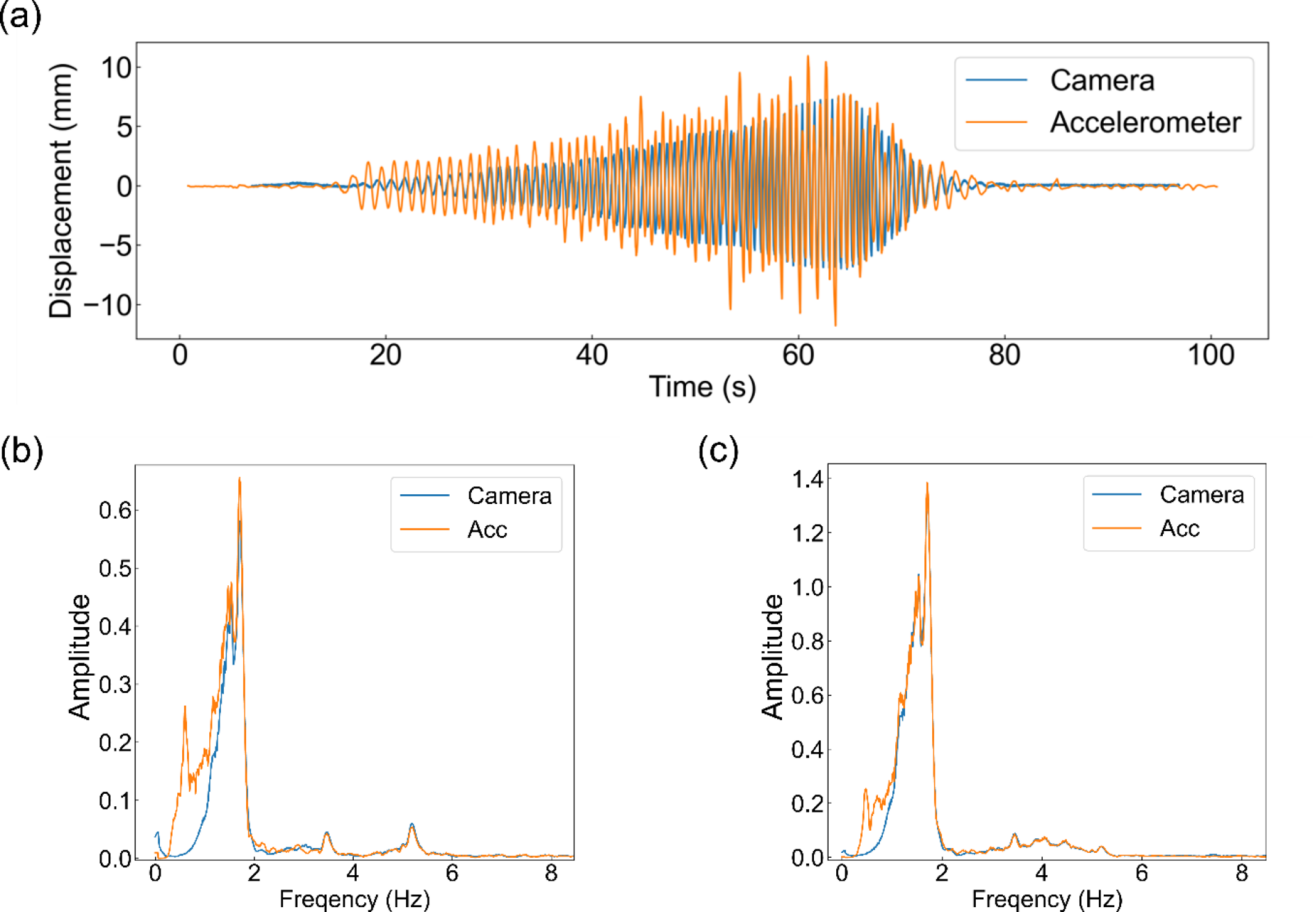
Comparison of the interstory drift data of X direction obtained by the accelerometers and the camera of the fabricated device. (**a**) Measured raw data. (**b**) Amplitude spectrum of X direction. (**c**) Amplitude spectrum of Y direction.

Figure 10 shows the results of the interstory drift measurements using the fabricated device and a higher-performance industrial camera during the shake table experiment. This indicates that almost the same signals were obtained for both. The LDS for reference could not be installed on the shake table. However, according to the results of the scaled-model experiment, an industrial camera can be used as a reference. Therefore, this figure indicates that the fabricated device can measure the interstory drift, similar to a reference. For comparison, Fig. 11 (a) shows the interstory drift measured using the accelerometer on the ceiling of the house. This indicates that the interstory drift obtained by integrating the acceleration twice does not match the reference value well. Figure 11 (b) and (c) show the amplitude spectra of the measured inter-story drifts. This indicates that the low-frequency components have large integration errors.

**Fig. 12:**
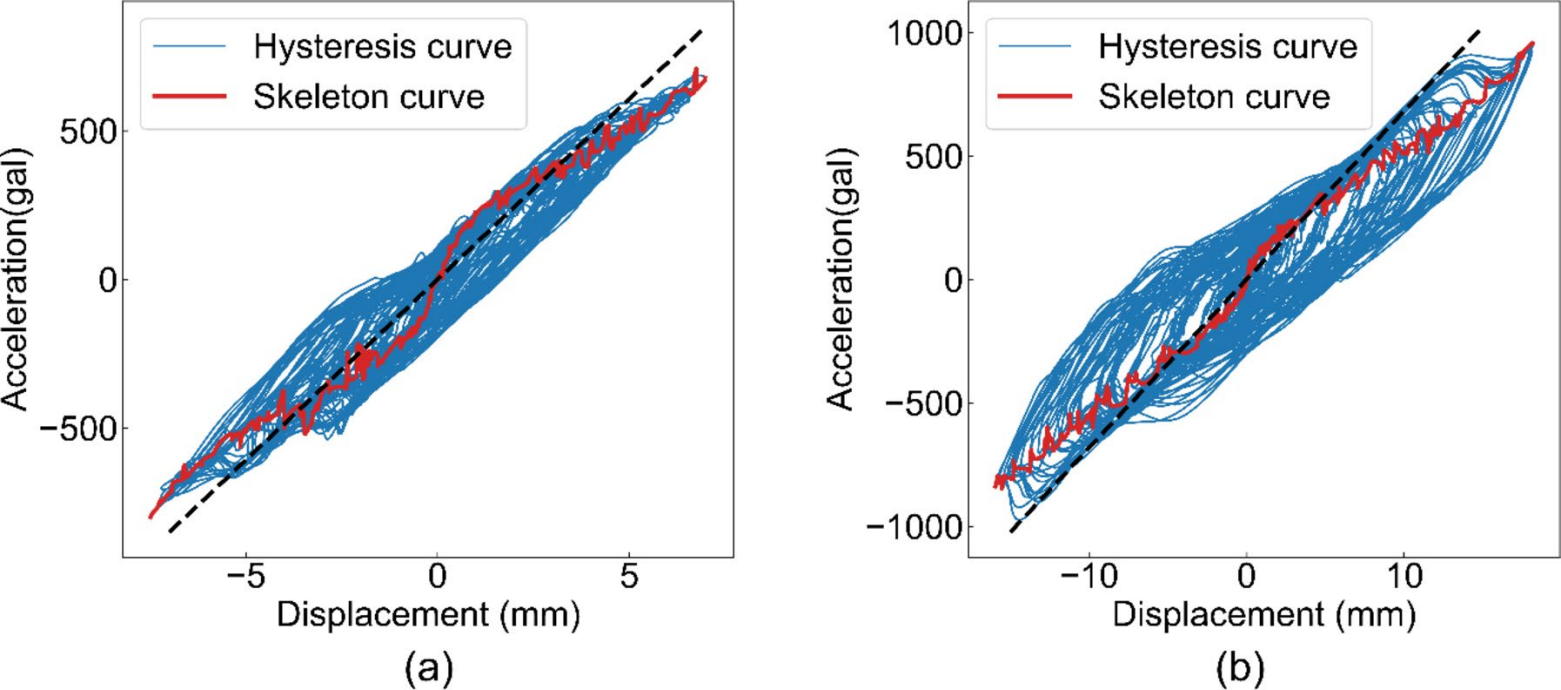
Measured restoring force characteristic by the fabricated device. (**a**) X direction, (**b**) Y direction.

The restoring-force characteristics are shown in Fig. 12. The dashed line represents the equivalent slope of the skeleton curve, calculated using the natural frequency. This indicated that the slope generally agreed with the skeleton curve. In addition, this figure shows that the skeleton curves indicated a change in the rigidity of the house. Specifically, the slope of the curve was high at low displacement and gradually decreased with increasing displacement. This corresponds to the known theory.

## Conclusion

In this study, a method to sense the restoring force characteristics using camera and accelerometer fusion was developed. The interstory drift was measured using a camera, and the response acceleration was calculated by adding the ground acceleration measured by the accelerometer and the interstory acceleration measured using the camera. These can be used to obtain the restoring-force characteristics of a house when assuming a single-mass system. This method does not require double integration of the acceleration because it measures the displacement directly; therefore, it has no integration errors such as shown in previous studies.

Experiments using the scale model showed that the restoring force characteristics could be measured with a measurement error of approximately 3% by combining a 100-fps camera and an accelerometer. In addition, upsampling to 300 fps suppressed the error by 1%, including when using a common camera with a frame rate of 40 fps. This method was also demonstrated using a shake table experiment in a wooden house. The prototype, fabricated using the camera and accelerometer, successfully measured the restoring force characteristics of a full-scale two-by-four timber-framed building.

Future studies will evaluate the performance of the device in various practical environments for social implementation. Factors such as ambient illuminance, the shape of the target ceiling surface, and the tilt of the ceiling or floor can affect the accuracy of our method. We will clarify the performance of our device in each actual situation. In addition, we will consider replacing the camera in the device with another sensor, such as a radar, which has an advantage in terms of privacy protection.

The proposed method is expected to be widely used because it does not require difficult operations such as synchronizing multiple devices, but only a small module. This method can quickly diagnose earthquake damage in detached wooden houses with installed modules, correctly determine whether evacuation is necessary, and reduce secondary damage from aftershocks.

## Data Availability

The datasets generated and analyzed during the current study are available from the corresponding author on reasonable request.
